# Single Cell Metabolomics: A Future Tool to Unmask Cellular Heterogeneity and Virus-Host Interaction in Context of Emerging Viral Diseases

**DOI:** 10.3389/fmicb.2020.01152

**Published:** 2020-06-03

**Authors:** Rajesh Kumar, Mayukh Ghosh, Sandeep Kumar, Minakshi Prasad

**Affiliations:** ^1^Department of Veterinary Physiology and Biochemistry, Lala Lajpat Rai University of Veterinary and Animal Sciences, Hisar, India; ^2^Department of Veterinary Physiology and Biochemistry, RGSC, Banaras Hindu University, Mirzapur, India; ^3^Department of Veterinary Surgery and Radiology, College of Veterinary Sciences, Lala Lajpat Rai University of Veterinary and Animal Sciences, Hisar, India; ^4^Department of Animal Biotechnology, Lala Lajpat Rai University of Veterinary and Animal Sciences, Hisar, India

**Keywords:** single cell metabolomics, virus-host interaction, emerging viruses, mass spectrometry, imaging

## Abstract

Viral emergence is an unpredictable but obvious event, particularly in the era of climate change and globalization. Efficient management of viral outbreaks depends on pre-existing knowledge and alertness. The potential hotspots of viral emergence often remain neglected and the information related to them is insufficient, particularly for emerging viruses. Viral replication and transmission rely upon usurping the host metabolic machineries. So altered host metabolic pathways can be exploited for containment of these viruses. Metabolomics provides the insight for tracing out such checkpoints. Consequently introspection of metabolic alteration at virus-host interface has evolved as prime area in current virology research. Chromatographic separation followed by mass spectrometry has been used as the predominant analytical platform in bulk of the analyses followed by nuclear magnetic resonance (NMR) and fluorescence based techniques. Although valuable information regarding viral replication and modulation of host metabolic pathways have been extracted but ambiguity often superseded the real events due to population effect over the infected cells. Exploration of cellular heterogeneity and differentiation of infected cells from the nearby healthy ones has become essential. Single cell metabolomics (SCM) emerges as necessity to explore such minute details. Mass spectrometry imaging (MSI) coupled with several soft ionization techniques such as electrospray ionization (ESI), laser ablation electrospray ionization (LAESI), matrix assisted laser desorption/ionization (MALDI), matrix-free laser desorption ionization (LDI) have evolved as the best suited platforms for SCM analyses. The potential of SCM has already been exploited to resolve several biological conundrums. Thus SCM is knocking at the door of virus-host interface.

## Introduction

“The risk from viruses is an unanswered question.” is such a meaningful quote with multi-directional implications. The enormous genetic diversity of existing viruses and unpredictability of viral behavior complemented by inadequacy of knowledge about global virome database always poses potential threats over the entire living world to acquire infection from any emerging or re-emerging viruses. Rapid climate change, increased traveling in the era of globalization and frequent mingling of sylvatic and urban lifecycle have also potentiated the viruses for genetic undulation, emergence and re-emergence to become pandemic at occasions (Devaux, 2012). Keen introspection regarding the virus-host interaction is a primordial aspect to reduce the knowledge gap and prepare the answer for the upcoming viral outbreaks. Exploitation of the highly specific nature of the virus-host interaction as well as the virus-cell interaction which are key for completion of viral life cycle and their transmission can offer crucial information to combat emerging viral infections. The fundamental cellular components required for viral replication and the host restriction factors to counter them can be of immense value to decide the checkpoints and therapeutic targets for customizing effective anti-viral mechanisms (Lassen, 2011). Single cell omics tools although in its budding age, still have enough penetration and resolution to introspect at the cellular and sub-cellular level for elucidation of such useful information. Progress in this arena is going on apace which will enable to explore the changing pattern of the viral interactions in cellular heterogeneity under variable ecosystem and host subjects. Suitable theranostics and effective vaccine of broad host specificity by unveiling the common pathways of viral interaction are the need of the hour to counter the ever-ending viral heterogeneity and future pandemics. The security of “One Health” concept in current scenario depends immensely on symbiotic development and convergence of several high throughput techniques such as but not limited to single cell omics, nanotechnology, artificial intelligence and various computational platforms (Minakshi et al., 2019a). The present review will focus toward various developments in single cell metabolomics tools and their few existing and mostly the probable future applications in unveiling the host virus interactions to acquire new armory in the arsenal for combating the emerging viral diseases.

## Single Cell Metabolomics (SCM)

“Omics” technologies pertain to the entire sets of molecules expressed at cellular or tissue level, or within an organism under specific set of condition. However cellular heterogeneity in multicellular organisms is often masked in pooled analyses. There the importance of single cell omics can be apprehended because it holds the potential to identify several targets expressed within a single individual cell or a cell population of interest individually at a specified time, or analyze handful of parameters in the same cells over time (Minakshi et al., 2019a). The enormous utility and popularity of omics tools have left almost no stone untouched in any of the biological research arena; the case is also not unlike in unveiling virus host interaction. As single cell omics tools are mostly in its naïve stage, thus ample evidences of their application at virus host interface is currently lacking but surely not too far in future which can be easily predicted from the bulk of omics interventions in terms of genomics, transcriptomics, proteomics, metabolomics and interactomics in the current perspective. Other single cell omics techniques have been employed to explore virus-host interaction in recent years (Dhillon and Li, 2015; Labonté et al., 2015; Drayman et al., 2017, 2019; Zanini et al., 2018; Rosenwasser et al., 2019; Russell et al., 2019; Wang S. et al., 2019). However SCM techniques are yet to start contributing significantly in understanding virus-host interaction.

Metabolites are smaller molecules, usually of lesser than 1.5 kD in size reflecting various cellular pathways which include sugar, lipids, glycolytic products (pyruvate, lactate, etc.), phosphate compounds (AMP, ADP, and ATP etc.), xenobiotics etc., but generally excludes nucleic acids, minerals, and salts (Minakshi et al., 2019a, b). They are arguably the terminal product of the basic central dogma process and provide the most nascent information about the phenotype. Metabolomics refers to the study of entire set of metabolites at a specific time-point. It is advantageous in understanding host-virus interaction in terms of generating immediate information regarding the exact downstream effects and ultimate fates of the analytes. It can yield a snap shot of dynamic and vulnerable host metabolites in response to its viral counterpart modulating each pathways at a particular infection stage. A recent flush of literature in metabolomics targeting virus-host interaction vividly justifies the utility of such analysis in current context (Table 1). Precise sampling methods accompanied by robust and high throughput analytical platforms such as nuclear magnetic resonance (NMR), receptive separation-based methods such as capillary electrophoresis (CE) or liquid chromatography (LC) and gas chromatography (GC) coupled with mass spectrometry (MS), and fluorescence-based techniques have delivered significant precision to “metabolomics.” Parallel progress in metabolite databases and bioinformatics programs have also assisted to generate an array of valuable information in a single go to match the expectations (Minakshi et al., 2019a). However single cell metabolomics (SCM) is obviously beneficial over population study alike other omics techniques to obtain specific temporal information regarding cell differentiation and division, communication, interaction with surroundings, stress responses including stage-specific metabolomic changes during viral infection (Strzelecka et al., 2018).

**TABLE 1:** List of application and salient findings of metabolomics techniques in emerging viral diseases.

Sl. no	Virus	Sample	Technique	Finding	References
1.	DENV	Plasma	^1^H nuclear magnetic resonance (NMR)	Low-density lipoprotein (LDL) and very-low-density lipoprotein (VLDL) decreased in all patient was same for lipoprotein in severe cases indicating importance of lipid metabolism in progression, Further Level of glutamine can have prognosis value	El-Bacha et al., 2016
2.	Early febrile, defervescence, and convalescent stages DENV	Serum	Liquid chromatography- mass spectrometry (LCMS)	Sixty differential metabolites, mainly affecting pathways of fatty acid biosynthesis and β-oxidation, phospholipid catabolism, steroid hormone pathway had been identified. further study revealed most metabolome changes are reversible and highest change occurs at early febrile including increase of serum inosine, cortisol, GlcCer	Cui et al., 2013
3.	H_1_N_1_ virus	Serum	UPLC - LCMS	Array of metabolites were present differentially pre and post treatment regime including 30 metabolites which can be potential biomarkers affecting multi-metabolic pathways including immune system arachidonic acid	Chuanjian et al., 2012
4.	DEN-1, DEN-2, DEN-3, and DEN-4	EA.hy926 cell line	H NMR and MS	Found alteration in metabolites like amino acids, dicarboxylic acids, fatty acids, and organic acids at significant level related to the tricarboxylic acid (TCA) cycle	Grace et al., 2010
5.	Dengue	Urine	^1^H NMR	Various compounds like organic acids, betaine, valeryl glycine, myo-inositol and glycine and amino acids were distinctly varied in patient while reduction in N-acetylglutamic acid, creatinine, myo-inositol, cre-atine phosphate, succinic acid, citrate, and 3-hydroxy-3- methylglutarate was also reported indicating amino acid metabolism, tricarboxylic acid intermediates cycle and β-oxidation of fatty acids were altered hence proposed highly predictive model for diagnosis by urine	Nurul et al., 2017
6.	Chikungunya and dengue	Serum	NMR spectroscopy	Fever, inflammation, energy deprivation and joint pain pathways were effected indicated by study	Shrinet et al., 2016
7.	Hepatitis C virus	Urine samples	NMR	66 patient study revealed metabolic fingerprint’ with 94% sensitivity and specificity of 97% with accuracy of 95% for non-invasive diagnosis	Godoy et al., 2010
8.	Hepatitis B	Serum	GC-MS	In a study on 47 patient a total of 25 metabolites identified were glyceric acid, cis-aconitic acid and citric acid were diagnosed with 93.62% predictive accuracy for diagnosis	Mao et al., 2008
9.	Hepatitis E	Plasma and urine	H NMR	Used for diagnosis 99.1 and 92.9% accuracy with reported various significant changes of metabolites indicating alteration of glycolysis, tricarboxylic acid cycle, urea cycle, and amino acid metabolism pathways more particularly amino acid metabolism, as aromatic amino acid (AAAs)	Munshi et al., 2011
10.	HIV/AIDS patients	Plasma, urine, and saliva	NMR	Twenty-six metabolites expressed differentially between ART-naïve patients and those receiving therapy leading to significant alternations in 12 metabolic pathways were have at least 2 metabolites are differentially regulated further proposed urinary Neopterin, and plasma Choline and Sarcosine could be used as metabolic biomarkers of HIV/AIDS	Munshi et al., 2013
11.	HIV-1	Serum	H NMR	34 HIV-1 positive 29 with treatment and 5 no treatment, techniques could distinguish between HIV-1 positive/AIDS patients	Hewer et al., 2006
12.	HIV	Oral ringe	LC/MS/MS and GC/MS	In study on 12 HIV-infected and 12 healthy individual, 98 identifiable and 85 non-identifiable metabolites identified of which 12 increased and 15 decreased altering carbohydrate biosynthesis and degradation pathways	Mahmoud et al., 2013
13.	HIV	Serum	Fourier transform Infrared (FTIR) spectroscopy	Quick method to diagnose	Lungile et al., 2014
14.	DENV-4	Serum	LC MS/MS	Three precursors of Platelet Activation Factor (PAF), three Phosphatidylcholine derivatives, four triglycerides as characteristic for DENV infection was identified affecting synthesis of PAF, remodeling inflammatory pathways, immune response autophagy process	Melo et al., 2018
15.	H1N1 influenza virus	Lung tissue samples from mice	LCMS	Total of 4,552 m/z ions (metabolic features) having 549 metabolites of 25 different pathways detected of which 49 features vary differentially including 10 decrease including methylniacinamide (metabolic product of niacin and Trp), pyrroline-5-carboxylate, amino acid asparagine, and hexaprenyl hydroxybenzoic acid and 39 increases includes cytidine, cytosine), a dipeptide breakdown product of protein cross-linking (_-glutamyl-lysine), kynurenic acid (Trp metabolite), and the essential amino acid, threonine in disease condition	Joshua et al., 2016
16.	Influenza	Serum, lung tissue and bronchoalveolar lavage fluid (BALF) of t 0, 6, 10, 14, 21, and 28 days post infection (dpi)	LC MS	More than 100 differential metabolites recorded, some indicating role of surfactant system in etiology, growth and control of virus	Cui et al., 2016b
17.	Influenza A virus	A549 and AGS cell line	GC/MS	AGS cell line might be more susceptible, Fatty acid biosynthesis and cholesterol metabolism as the target as emphasized by 12 and 10 metabolites differential expression.	Lin et al., 2010
18.	Nfluenza-associated encephalopath	Cerebrospinal fluid (CSF)	FT-ICMS	Two molecular weights, 246.0092 and 204.061 can be used for diagnose influenza with convulsion. It may be C_6_H_13_NaO_6_, C_6_H_14_O_6_(possibly mannitol), or C_12_H_12_OS	Kawashima et al., 2006
19.	H3N2 influenza infection or dengue fever	Serum	LC-MS	Comparison between two virus metabolome revealed 26 differential metabolites involved in purine metabolism, fatty acid biosynthesis and β-oxidation, tryptophan metabolism, phospholipid catabolism and steroid hormone pathway	Cui et al., 2017a
20.	DENV serotype 2 infection at 0, 3, 7, 14, and 28 days postinfection	Humanized mice serum	LC-MS	Forty-eight differential metabolites were identified altered significantly indicating changes various pathways in pyrimidine metabolism, fatty acid β-oxidation, phospholipid catabolism, arachidonic acid and linoleic acid metabolism, sphingolipid metabolism, tryptophan metabolism, phenylalanine metabolism, lysine biosynthesis and degradation, and bile acid biosynthesis	Cui et al., 2017b
21.	Chronic hepatitis B	Serum	Targeted LCMS	In study n Sixty-nine chronic HBV patients and 19 healthy controls given 314 metabolites where decrease hijacking of the glycerol-3-phosphate–NADH shuttle occurs while Oxylipins were increased	Schoeman et al., 2016
22.	Human cytomegalovirus (HCMV) and herpes simplex virus type-1	Fibroblast and epithelial host cells	LC-MS	80 metabolites detected reveled metabolic changes were conserved in different type of host cell	Vastag et al., 2011
23.	Hepatitis C	Huh-7.5 cells (human hepatoma cell line)	LC-MS	Identify 272 lipids at different concentration and time variations, necessary for virus replication, downstream assembly and secretion	Diamond et al., 2010
24.	Influenza vaccine strain	MDCK cells	LCMS	0–12 h post infection study average time of metabolic switch for all 29 metabolites included was 11.6 h	Ritter et al., 2010
25.	Human cytomegalovirus (HCMV)	Human fibroblasts	LCMS	63 different intracellular metabolites at multiple times of infection were identified	Munger et al., 2008
26.	Adenovirus	Breast epithelial cell line MCF10A		Product E4ORF1 localizes to the nucleus and binds the transcription factor glutaminase critical enzyme for HIV, Influenza & adenovirus replication	Thai et al., 2014
27.	Rhinovirus (RV)	HeLa and Fibroblasts Female C57BL/6 J mice	UPLC-MS/MS	Showed metabolites associated with glycogenolysas glucose deprivation from medium and via glycolysis inhibition by 2-deoxyglucose (2-DG) potently impairs viral replication, glucose and fatty acid uptake were up-regulated after 1.5 h in primary human fibroblasts. Further revealed a specific fingerprint of RV infection can be use for diagnosis	Guido et al., 2018
28.	HIV	CD4^+^ T cells and macrophages U937 cell line	FACS sorting and LC- MS/MS	A total of 66 metabolite identified, infected CD4^+^ T cells In macrophages substantial reductions in glucose uptake and steady state glycolytic intermediate, Showed cell types exhibit very different metabolic outcomes	Hollenbaugh et al., 2011
29.	HIV-1 and HIV-2	Monocyte-derived macrophages (MDMs)	LC–MS/MS	Macrophage infected with both type of virus have similar metabolic profiles in relation to glycolysis and TCA cycle	Hollenbaugh et al., 2016
30.	Hepatitis C virus	Whole cell extracts and subcellular compartments of HEK293T cells	LC-MS/MS	Membrane lipids, especially cholesterol and phospholipids, accumulated in the microsomal fraction in HCV-infected cells, phosphatidylcholines and triglycerides with longer fatty acyl chains were higher so the utilization of C18 fatty acids	Hofmann et al., 2018
31.	Zika virus (ZIKV)	Serum	ESI-MSMS	79 metabolites analysis revealed Glycosphingolipids Ganglioside GM2, in the nervous tissue was elevated and proposed as marker so was series of phosphatidylinositols. Study also revealed upregulation of Angiotensin and Angiotensin I., Furthermore it also described first time that lipids for ZIKV infection are described	Melo et al., 2017
32.	Respiratory Syncytial Virus (RSV) is	Lung epithelial cellsA549 cells, human alveolar epithelial like cells	LCMS	122 oxylipins were identified and analyzed	Schoeman, 2020
33.	Respiratory Syncytial Virus (RSV) is	Lung epithelial cells A549 cells, human alveolar epithelial like cells, BALB/c mice bronchoalveolar lavage and infant nasopharyngeal secretions	LCMS	A total of 74 amine metabolites were identified indicating down-regulate key anti-oxidant enzymes reveled by the fact compromised sulfur metabolism, glutathione and taurine while increased cysteine and cystathionine	Schoeman, 2020
34.	Hepatitis B virus (HBV)		Targeted metabolomics approach on serum	Reveled reduced glycerophospholipids and increased plasmalogen Species in immune tolerant phase indicating viral hijacking of the glycerol-3-phosphate-NADH shuttle while in chronic condition, increased very long chain triglycerides, citrulline and ornithine was observed in study over 88 samples where a total 92 significant metabolites were identified	Schoeman, 2020
35.	HIV-infected women	Effect on infant, cord blood plasma	LC-MS targeted metabolomics analyses	Increased triglyceride, oxidized lipids, pro-inflammatory lysophospholipids, 2 diacylglycerols and 32 triglycerides and decreased phospholipid was reveled on analysis of 12 positive HEU infants and 15 HUU infants negative infant	Schoeman, 2020
36.	Human cytomegalovirus (HCMV)	Human fibroblasts, microarray	LC-MS/MS	Several different metabolites were identified at various time intervals and related with glycolysis, the citric acid cycle	Munger et al., 2008
37.	Dengu	Blood plasma	GCMS	Profiling of esterified fatty acids as biomarkers revealed saturated fatty acids, C14:0, C15:0, C16:0 and C18:0 decrease significantly	Khedra et al., 2014
38.	Dengue, hepatitis B and hepatitis C	Serum	LC-TQ-MS	Thirty-five phospholipids were identified and characterized in lipomoic study and 28 PLs were determined to be significantly different in DF, HBV, and HCV	Khedr et al., 2016
39.	Infectious spleen and kidney necrosis virus (ISKNV)	Chinese perch brain cells (CPB)	UHPLC-Q-TOF/MS	Metabolic Shift from Glucose to Glutamine was observed	Fu et al., 2019
40.	DENV	Blood	GCMS	Sixty differential metabolites involved in pathways included fatty acid biosynthesis and b-oxidation, phospholipid catabolism, steroid hormone pathway with temporal change with phospholipids and sphingolipids as prognosis marker since showing association with lymphocytes and platelets numbers were identified	Cui et al., 2013
41.	Dengu	Serum samples from dengue patients from Nicaragua and Mexico	HILIC-MS	306 metabolites differentiated between DF from ND37DHF/DSS and DF outcomes from Mexican, and 191 metabolites differentiated DF from ND outcomes and 83 differentiated DHF/DSS and DF outcomes were identified further data analysis revealed that 65 metabolites panel can predicted dengue disease outcomes	Voge et al., 2016
42.	Dengu	116 dengue patients serum	LCMS	20 metabolites differentially Enriched while further analysis revealed that Serotonin can indicate Severe Dengue in the Early Phase, while Combining serotonin and IFN-γ improved the prognosis	Cui et al., 2016a
43.	Newcastle disease virus	DF-1 cells	UHPLC-QTOF-MS	Identified 305 metabolites with significant changes mostly belonging to belong to the amino acid and nucleotide metabolic pathway	Liu et al., 2019
44.	Ebola	Plasma lipidome	LC-MS/MS	EVD survivors and fatalities from Sierra Leone, Samples reveled insight on role of lipome profile in viremia, liver dysfunction, apoptosis, autophagy, and general critical illness where a total of 423 lipid were identified, further data study indicates significant differences of 30.6% & 59.4% between fatalities vs. initial diagnosis survivor and recovered survivor. Further deep digging between healthy and survivor indicates 50.7% altered lipid profiles	Kylea et al., 2018
45.	Ebola virus	Plasma of healthy, survivor and fatal	LCMS	Acute reduction in plasma free amino acids (PFAAs), in EVD fatalities was observed, plasma lipid species was also significantly altered	Eisfeld et al., 2017
46.	Ebola Virus and Marburg Virus	Licorice root	Mass spectrometry HRMS and MS/MS analysis	Identified two compounds 18β-glycyrrhetinic acid and licochalcone A, can bind with virus nucleoprotein	Fu et al., 2016
47.	Lassa virus (LASV)	Serum from febrile patients of 28 female and 21 male patient	LCMS	3100 and 6900 different spectral features were obtained, in fatal cases analysis indicates nearly all PAFs or PAF-like molecules were present in lower amounts same was true for D-urobilinogen and I-urobilin a hem breakdown product, whereas 7-methylinosine was elevated out of 153 lipid compound identified, half of them were in lower amount	Gale et al., 2017
48.	Vesicular stomatitis virus (VSV)	BHK cells		Single-Cell Kinetics in presence of Defective interfering particles (DIPs)	Akpinar et al., 2015
49.	Hepatitis B virus	Serum	Both GC-MS and LC-MS analysis	A total 427, 382, and 243 mass feature were identified changing significantly between different group HBV-cirrhosis and control, HCC and control as well as HCC and HBV respectively, of which 14 metabolites consistently altered in HBV-cirrhosis and HCC patients	Schoeman, 2020
50.	HIV-1	Macrophages	LCMS	Virus – macrophage interaction reveled increase in macrophage fumarate, glucose uptake	Datta et al., 2016
51.	Influenza A virus (IAV)	A549 Cells	UHPLC–Q-TOF MS	In study on first cycle, replication the metabolic profile which includes 50 differentially expressed metabolites at different time-lag, indicate hijacking of cell metabolism for its own replication, and affecting innate immunity and affecting nearly 30 pathways	Tian et al., 2019
52.	Classical swine fever (CSF)	Serum	UPLC/ESI-Q-TOF/MS	Ten Virulent form infected days 3 and 7 post-infected piglet and 10 healthy study revealed disturbance in tryptophan catabolism and the kynurenine pathway	Gong et al., 2017
53.	HIV	Cerebrospinal fluid of rhesus macaques	LCMS	3,687 features were observed of which twelve metabolites were elevated	William et al., 2008
54.	Norovirus	RAW 264.7 cells	LCMS	Assay reveled glycolysis, OXPHOS, glucuronic acid, pentose phosphate pathway (PPP), adenosine catabolism increased, was supported by enhanced fructose-bisphosphate, 2- and 3-phosphoglycerate, dihydroxyacetone-phosphate, 6-phosphogluconate, citrate/isocitrate, malate inosine-monophosphate (IMP), hypoxanthine and xanthine, uridine tri-phosphate (UTP), UDP-glucose, and UDP-D glucuronate	Karla et al., 2018
55.	HIV-1	CD4 + T cells	LC-MS/MS, NMR analysis, Flow cytometry	Glutamine concentrations are elevated, glutamic acid secretion increase, Intracellular amino acid content was also changing	Hegedus et al., 2017
56.	Murine cytomegalovirus (MCMV)	Bone marrow derived macrophages (BMDM)	Level	Glycolysis, TCA cycle and urea cycle and fatty acid metabolism alterations was evidenced by metabolomics study indicating modulation of host environment and immunity	Konstantinos, 2017
57.	MERS-CoV	Calu-3 cells	LC-MS	Lipid metabolism chemical most effected	Yuan et al., 2019
58.	Ebola viruses	Compound of Piper nigrum		7 compound including HJ-1, HJ-4 and HJ-6) strongly promoted formation of large NP oligomers and reduced protein thermal stability	Wang Z. et al., 2019
59.	H9N2 (avian), H6N2 (avian), and H1N1 (human	B lymphoblastoid cells	GC/MS	Volatile organic compounds analysis revealed production of esters and other oxygenated compounds	Aksenov et al., 2014
60.	Influenza	Male ferrets nasal washes	GC-MS	Oseltamivir treatment showed different metabolome behavior in cell	David et al., 2019
61.	H1N1 influenza	Mice lung	LC MS	396 metabolites significantly associated with inflammatory Cytokines were identified, Further study of data indicates associations with vitamin D, E and purine, metabolism with inflammatory molecule IL-1_, TNF-α, and IL-10. Anti-inflammatory cytokine IL-10	Chandler et al., 2016.
62.	H1N1 pneumonia	Plasma, culture	^1^H nuclear magnetic resonance spectroscopy and gas chromatography-mass spectrometry	42 patients and control metabolome revealed various molecules for diagnosis and Prognosis where more than 88 metabolites had been observed significantly altered between, healthy, survivor, non-survivor patient	Banoei et al., 2017
63.	H7N9 Infection	Serum samples	UPLC-MS	In 33 sample of healthy and diseased study Metabolome study revealed that disease severity and metabolism change of fatty acid are related	Sun et al., 2018
64.	Hepatitis B virus	Serum	Untargeted metabolomics (UPLC-MS)	33 potential biomarkers were identified of which 25 regulated by syringing were involved in 8 metabolic pathways, the data further suggest a potential of Amino acid metabolism as target for the treatment	Jiang et al., 2020

Research related to virus host interaction and information regarding susceptibility, virulence, mechanisms of gene regulation and metabolic pathway modulation under variable environmental factors such as temperature, humidity, light color, intensity and also radiation etc. are mostly lacking that may be useful to combat the incidences of outbreaks from novel viral emergence. Although metabolomics has found its value in virology research but mostly in population study whereas SCM intervention is almost yet to be started to introspect virus-host interaction and is reached upto the level of cell lines only, may be due to the hardship of SCM in terms of single cell isolation and handling, structural diversity and rapid metabolite turnover, lack of amplification opportunity, sensitivity and repeatability of the analytical platforms etc. (Table 1; Minakshi et al., 2019a). However it’s not tough predictions that no longer will SCM be left incumbent considering the worth of information it generates and its ample application in the current context will be reflected in near future.

## Sample Preparation Methods for Single Cell Isolation for SCM Analysis

**FIGURE 1:**
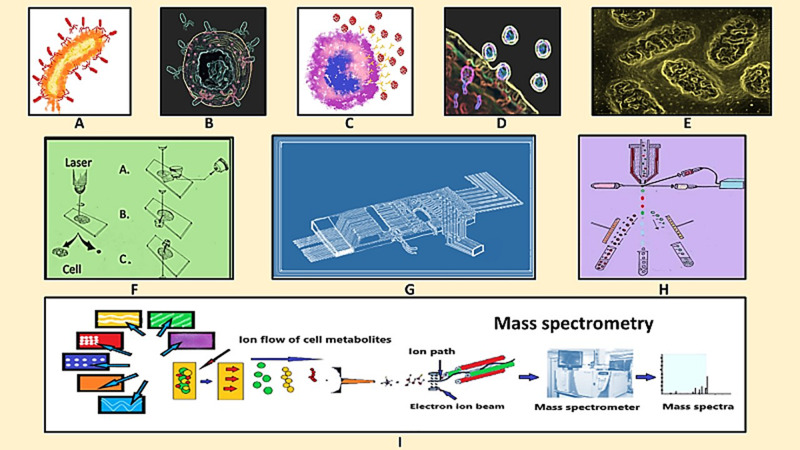
Schematic diagram of virus-host interaction, single cell isolation and single cell metabolomics analysis. **(A)** Virus infecting the host cell, **(B)** virus-macrophage interaction, **(C)** virus encounters immune cell, **(D)** virus-host cell interaction, **(E)** metabolic changes at sub-cellular level, **(F)** Single cell isolation by laser microdisection, **(G)** microfluidic chip for single cell isolation, **(H)** fluorescence based cell sorting, and **(I)** mass spectrometry for single cell metabolomics analysis.

The most common hurdle in SCM analyses is the isolation of single target cell followed by its careful handling and delivery to the downstream analytical platforms because it is directly reflected over the precision of SCM data. Limiting dilutions, manual cell-picking by micromanipulator and laser micro dissection are the common manual methods having relatively low throughput yet applied for single cell analyses where as fluorescence-based cell sorting, high-density microarrays and microfluidics are the most popular automatic high throughput single-cell isolation techniques employed for SCM (Gross et al., 2015; Minakshi et al., 2019b; Figure 1 and Table 2). High cost involvement is a major limitation associated with all of these high end techniques.

**TABLE 2:** Single cell isolation Techniques and platforms for Single cell metabolomics analysis to elucidate Virus-host interaction.

Techniques/Platform	Advantage	Limitations
**CELL ISOLATION TECHNIQUES**		
Limiting dilutions, manual cell-picking	Very simple, no advance instrumentation, inexpensive	Low throughput, Issue of multi-step handling
Laser capture micro dissection	No marker required	Low throughput, cost-intensive, laser-induced quenching issue
Fluorescence-based cell sorting (FACS) & Magnetic -based cell sorting (MACS)	High throughput, multiplexing is possible	Need specific marker for sorting target cell, preferred for cells suspended in liquid media, Laser or Magnetic field may affect cellular metabolites, cost-intensive
Microfluidics	High throughput, minimum sample handling, all steps from quenching to isolation in one go, compatible with multiple analytical platform	Need to design specifically as per requirement i.e., custom made, cost-intensive
Aptamer-based	Living *cells can be isolated*	Need knowledge of structural conformation
Single-cell printing technique	Living *cells can be isolated*	Still in initial stage of development, cost-intensive
*Direct sampling by electrospray ionization (ESI) Tip*	Minimum sample handling, Living cell and its organelle can be imaged, high throughput	Costly, skill-intensive
**ANALYSIS PLATFORM**		
Mass Spectrometry like Capillary electrophoresis-coupled-mass spectrometry (CE-μESI-MS), High-Resolution Mass Spectrometry (CE-ESI-HRMS), Matrix assisted laser desorption/ionization (MALDI) MS	Highly sensitive, Label free, for targeted and untargeted metabolites, both qualitative and quantitative metabolomics can be performed	Cost-intensive, advanced instrumentation required still cannot map all metabolites in one go
Gas chromatography Time-of-Flight mass spectrometry (GC-TOF-MS)	Highly accurate, sensitive	Mostly for volatile compounds
Mass Spectrometry Imaging: Matrix-Assisted Laser Desorption Ionization (MALDI)-MSI and Electrospray Ionization (ESI)-MSI	Highly sensitive, minimum sample handling, imaging of even sub cellular component is possible	Resource intensive, still in native stage of development
NMR	Highly accurate and robust than LC-MS	Low sensitivity to achieve penetration upto single cell level
Surface Enhanced Raman Scattering (SERS)/Vibrational Spectroscopy	Fast, label-free non-destructive method	specific spectra for metabolite are not reveled but only for spectroscopically active and concentrated metabolites
Fluorescence-based detection	For targeted metabolites	Limited multiplexing option, cannot cover all range; very few metabolites are autofluorescent and nanosensor probes exist for only a handful of specific small mole
Matrix-free laser desorption ionization (LDI) mass spectrometry imaging methods: Desorption Ionization on Silicon (DIOS)-MSI, surface-assisted laser desorption ionization (SALDI)	Highly sensitive, excellent coverage	Cost and Resource intensive, still at early age of development

Fluorescence-activated cell sorting isolates the target cells specifically labeled with antibody coupled with a fluorescent tags. This technique prefers the samples that are naturally suspended in liquid media, such as blood, bone marrow cells, semen, ovum, secretions, yeast, protoplast, bacteria, and viruses (Ciuffi et al., 2016; Khalil et al., 2017; Janssens et al., 2018; Singh, 2019). Time-lapse fluorescence imaging is a similar type of fluorescence based single cell analysis technique used for monitoring of cell virus interaction (Drayman et al., 2017).

Immune-magnetic separation using antibody-coated magnetic beads or attached with other ligands for capturing the target cells is also an important single cell isolation method. Dynabeads or MACS (Dynabeads Magnetic Separation Technology) employs such isolation principle (Miltenyi et al., 1990).

Aptamer-based cell isolation relies upon the precise binding of aptamers (single-stranded oligonucleotides, DNA or RNA) to its target using structural conformation and ease the isolation of single cells (Guo et al., 2006; Hu et al., 2016; Delley et al., 2018; Chen, 2019; Kacherovsky et al., 2019). Fluorescent nanodiamonds (FNDs) are diamond nano-crystals which have also been used for single cell labeling for cell tracking and illuminating the cellular processes under different conditions (Prabhakar et al., 2017; Claveau et al., 2018).

Single-cell printing technique like “Block-Cell-Printing” is another example of high throughput single cell isolation technique yet to find ample application (Gross et al., 2013; Zhang et al., 2014; Schoendube et al., 2015).

Microfluidics or Lab-on-a-Chip devices provide excellent opportunity for simultaneous isolation of target single cells along with its handling and manipulation followed by SCM suitable platforms with high end automation (Warrick et al., 2016). Several analytical platforms such as electrophoresis, optical spectroscopy, fluorescence based imaging, or mass spectrometry are compatible with microfluidics based cell isolation. Soft lithographic fabrication onto polydimethylsiloxane (PDMS), glass or silicon chips provides the base of microfluidic devices and hydrodynamic or gravity-driven flow of cells are used to capture the target cells. Microfluidics device installed on a fluorescence microscope has yielded valuable information regarding the kinetics of viral infection in single cells and variability of cellular factors regulating the viral replication (Guo et al., 2017). Hybrid microfluidics using either active or passive approaches or their combination, integration of nano- and microfluidics, digital or virtual microfluidics are the emerging microfluidic platforms which are being recently introduced for high throughput and continuous single cell isolation avoiding the general limitations of the existing technique (Xu et al., 2016; Yan et al., 2016; Wang et al., 2017; Vanderpoorten et al., 2019).

Direct sampling through nano-electrospray ionization tips followed by mass spectrometric analyses method is gaining popularity in recent times due to its minimum sample requirement and processing along with high throughput data generation. Pipette tip column electrospray ionization (PTC-ESI) or ballpoint electrospray ionization (BP-ESI) followed by simultaneous mass spectrometry or live single-cell video-mass spectrometry are the SCM platforms where the technique has been used (Tsuyama et al., 2008; Huang et al., 2012; Ji et al., 2016; Pu et al., 2018).

## Analytical Platforms for Single Cell Metabolomics

The precision and coverage of SCM analysis depends upon the “resolution” and “sensitivity” of the analytical platform. Although theoretically SCM analyze the entire set of cellular metabolites but more often it is restricted to qualitative or quantitative analysis of a cluster of metabolites under certain condition. “Targeted” for acquiring information regarding specific metabolite(s) or “untargeted” SCM approach for obtaining the maximum coverage can be chosen according to the requirement and selection of the analytical platform should be done accordingly. Mass Spectrometry Imaging (MSI)-based methods are arguably the most sensitive and thus preferred SCM platform. However, the compromised reliability, restricted quantitation option, and poor resolution for structural characterization are the certain limitations need to be taken care of. Other SCM platforms include but not limited to separation-based methods like capillary electrophoresis (CE), liquid chromatography (LC), or gas chromatography (GC) combined with MS or fluorescent tagging approach which are mostly used for “targeted SCM” due to the sensitivity issue (Minakshi et al., 2019a; Table 2).

## Separation-Based Methods Coupled With Mass Spectrometry

Capillary electrophoresis coupled with electrospray ionization and mass spectrometry (CE-μESI-MS) has been employed to analyze single 8-cell embryo of *Xenopus laevis* to depict more than 80 metabolites (Onjiko et al., 2015). Similarly CE-ESI-TOF-MS has explored over 300 metabolites from the neuronal cell of sea slug *Aplysia californica* (Nemes et al., 2013). CE-ESI-MS has quantitatively characterized over 15 anionic metabolites (nucleotides and derivatives) from *Aplysia* sensory neuron with the detection limits in <22 nM range (Liu et al., 2014). High-Resolution Mass Spectrometry (CE-ESI-HRMS) analyses of a single 16-cell stage embryo of *Xenopuslaevis*hasrevealed438 non-redundant protein groups by label-free quantification with a detection limit of ∼75 attomol (∼11 nm) (Lombard-Banek et al., 2016). Targeted automated SCM analysis of neuronal cell by a microchip electrophoresis-mass spectrometric method (MCE-MS) has quantitatively measured dopamine and glutamic acid content within individual cell with detection limits 8.3 and 15.6 nM for the metabolites, respectively. Although the introduction of this platform in virus-host interaction arena is pending but the mentioned evidences has clearly suggested about its potential in SCM analysis.

Nano-flow liquid chromatography-electro spray ionization mass spectrometry (LC-ESI-MS) based analysis has yielded 5 anthocyanins from 2-4picolitervolume of individual *Torenia hybrida* petal cell (Kajiyama et al., 2006). Gas chromatography Time-of-Flight mass spectrometry (GC-TOF-MS) analyses of *Mesembryanthemum crystallinum* epidermal bladder cells has identified 194 known and 722 overall molecular features which generate significant pathway information regarding metabolic alterations in salty environment (Barkla and Vera-Estrella, 2015). Thus chromatographic methods have great potential for SCM analyses in combination with MS platforms. Although not in single cell level but GC/MS based metabolomics technique has already been applied for introspection of fatty acid biosynthesis and cholesterol metabolism in A549 and AGS cell lines infected with influenza A virus yielding valuable information regarding susceptibility and cell differentiation in association with viral replication (Lin et al., 2010). Ultra-high-performance liquid chromatography/quadrupole time-of-flight tandem mass spectrometry (UHPLC-QTOF-MS) analyses has depicted significant changes in 305 metabolites during infection of DF-1 cells with the Herts/33 strain of New Castle Disease Virus (Liu et al., 2019). Gut metabolomic analyses of rhesus monkeys by GC-MS and LC-MS during gut virome alteration has been correlated with gut microbiome and the underlying role of metabolites like tryptophan, arginine, and quinine (Li et al., 2019). Although the penetration upto the single cell level is yet to be reached but the evidences are enough to ensure the utility and future of this platform for metabolomic analyses in virology research.

## Mass Spectrometry Imaging

Extreme sensitivity, vast coverage and the opportunity of label-free analysis has rendered MSI as one of the most preferred method of SCM analysis, particularly for untargeted approach. Improvement in resolution is going on in terms of reducing the laser spot area by lowering the diameter of optical fiber along with providing multiplexing option by integration of multiple mass analyzers. Matrix-Assisted Laser Desorption Ionization (MALDI)-MSI and Electrospray Ionization (ESI)-MSI are the two conventional platforms applied for SCM analyses (Figure 1).

Polarity-switching option is lacking in MALDI-MSI, it can be run in either ion mode at a time, multiplexing can be added subsequently for increasing the coverage. However, introduction of advanced matrices, such as 9-AA or silver, gold, or titanium and grapheme oxide nanoparticles, have increased the sensitivity by reducing background noise. Multiplex MSI platforms are equipped to directly explore individual intracellular metabolites, even their sub-cellular location irrespective of chromatographic separation. SCM analysis of by MALDI-MS operated in negative ion mode has revealed several metabolites such as ADP, ATP, GTP, and UDP-Glucose etc. from *Closterium acerosum* (Amantonico et al., 2010). Metabolic analysis of single Yeast cell by coupling microscale sampling and MALDI-MS at negative ion mode detection has elucidated information regarding some valuable metabolites such as ADP, GDP, ATP, GTP, and acetyl-CoA. The detection limit ranged from 5 to 12 attomoles (Amantonico et al., 2008). Pressure probe sampling (in picoliters) coupled with UV-MALDI MS has been applied for shotgun metabolite profiling of organic compounds live single plant cell (Gholipour et al., 2012). High spatial resolution MSI analysis of Maize root epidermal and cortex cell has elucidated several TCA cycle metabolites including their localization with spatial resolution improved to ∼5 μm (Hansen and Lee, 2018). Turkey gut microbiome has been introspected using MALDI-MS platform operated in positive ion mode with nanoparticle microarray and organic matrices (Hansen et al., 2018). Metabolomics variability during virus host interaction in bloom-forming alga *Emiliania huxleyi* has been evaluated by Plaque assay combined with several MSI techniques (Flow-probe-MSI, MALDI-MSI, UPLC-q-TOF MS, and LC-MS/MC for lipidomics, GC-MS for fatty acid methyl esters) (Schleyer et al., 2019). Fluorescence *in situ* hybridization (FISH) microscopy combined with high-resolution atmospheric pressure-MALDI-MSI of *Bathymodiolus puteoserpensis* epithelial cells has also been used to understand host-microbe symbiosis (Geier et al., 2019). Lipidomics profile of infected *Aedes albopictus* cells during Zika virus infection has been investigated using MALDI-MS platform to identify thirteen infection-specific lipid markers in the mosquitoes (Melo et al., 2016). Thus the mentioned evidences easily suggest about the utility and potential of MALDI-MSI based platform for SCM analyses and can be predicted more often application to evaluate host-virus interaction at single cell level.

Alike MALDI, Electrospray Ionization (ESI) is another important soft ionization technique where the analytes after ionization, evaluated by MS to facilitate metabolite characterization. ESI-MS is equipped with simultaneous detection at both positive and negative ion mode yielding enormous sensitivity. Live nano-ESI-LTQ-MS analyses of *Pelargonium zonale* leaf cell has depicted over16 metabolites from 1to 5pL of sample (Tejedor et al., 2009). Probe ESI mass spectrometry (PESI-MS) with tungsten probe of 1 μM diameter has detected several metabolites including 6 fructans, 4 lipids and 8 flavone derivatives in single *Allium cepa* cell (Gong et al., 2014). ESI-Q-TOF-MS operated in either positive or negative-ion mode has yielded status regarding several metabolites such as tryptophan, creatinine, dopamine, and glycine, and cofactors such as lactate and pyruvate to link energy metabolism and immunodeficiency in microgravity (Chakraborty et al., 2018).

Laser ablation electrospray ionization (LAESI) employs mid-infrared (mid-IR) laser to generate gas phase metabolites. Large plant cells or cluster of animal cells are preferred subjects for this platform but high sensitivity, little chemical background and a direct sample delivery to MS platform has made it a desired choice for SCM analysis. Metabolites from single cells or cluster of *Allium cepa* and *Narcissus pseudonarcissus* bulb epidermis and single eggs from *Lytechinus pictus* has been evaluated through LAESI to detect 35 prominent metabolites including anthocyanidins, flavonoids, and their glucosides depicting its potential in SCM analysis (Shrestha and Vertes, 2009). LAESI-MS coupled with ion mobility separation (IMS) has been described as capable of direct profiling and imaging of several biomolecules including metabolites from biological tissues and single cells (Etalo et al., 2018; Paglia and Astarita, 2020). LAESI coupled with a 21 tesla Fourier transform ion cyclotron resonance (21T-FTICR) has capable of elucidating isotopic fine structure of the biomolecules by direct MS analysis and imaging (Stopka et al., 2019).

Live Single-Cell Video-Mass Spectrometry using micro sampling method by video microscopy and micromanipulator guided gold-coated glass capillary nano-ESI tip along with Nano-ESI-Q-TOF platform has demonstrated good potential for SCM analysis (Lapainis et al., 2009; Hiyama et al., 2015).

## Non-Mass Spectrometric Methods

Next to mass spectrometry, NMR is a major tool for metabolite analyses. It is even robust and requires less sample processing than the other metabolomics tools. However requirement of large sample volume and the issue of sensitivity render it imperfect for SCM analyses rather it is preferred for tissue sample and biofluid introspection. For example continuous *in vivo* metabolism has been followed by using NMR (CIVM-NMR) coupled with high-resolution-magic angle spinning to elucidate branched-chain amino acid metabolism in human chronic lymphoid leukemia cells (Judge et al., 2019).

Fluorescence-based sensors are also being applied in targeted SCM analyses. CE with laser-induced fluorescence (LIF) detection has been employed for detection of chiral amino acids and neurotransmitters from mouse brain and neuron cells (Jakó et al., 2014; Qi et al., 2017). Capillary micro sampling MS with fluorescence microscopy has successfully identified 29 metabolites and 54 lipids from Human HepG2/C3Acancer cells (Zhang et al., 2018). Microchip electrophoresis coupled with LIF detection have been employed for identifying ethanol induced metabolite change in mice liver cells to depict elevated hydrogen peroxide along with depleted glutathione and cysteine level in the target cells (Li et al., 2016). However limited multiplexing option has restricted its application mostly within targeted approach.

Surface Enhanced Raman Scattering (SERS) employs laser in the visible, near-infrared, or near-ultraviolet range for label free detection of the metabolites within the cells (Shalabaeva et al., 2017). SERS has been applied for fast, label-free detection of *Chlamydia trahomatis* and *Neisseria gonorrheoae* along with associated extra-cellular metabolite changes (Chen et al., 2018). Raman spectroscopy is a non-destructive method for metabolite analysis but only few functional groups emit detectable Raman signals reducing the applicability of the method for SCM analyses (Smith et al., 2016). Recently, Raman micro-spectroscopy using graded X-ray doses has successfully uncovered several nucleus and cytoplasmic specific metabolic features from single SH-SY5Y human cancer cells (Delfino et al., 2019).

## Future SCM Techniques

Secondary Ion Mass Spectrometry Imaging with spatial resolution capacity at the range of 50–100 nm is a potential future tool for SCM as well as sub-cellular metabolite analyses (Svatos, 2011; Kollmer et al., 2012; Gilmore et al., 2019). The matrix-free laser desorption ionization (LDI) methods include Desorption Ionization on Silicon (DIOS)-MSI which is a variant of surface-assisted laser desorption ionization (SALDI), both use soft ionization of analytes followed by MS analyses. LDI-MS platform employing HR-MS along with Orbitrap FT-MS has been used for SCM analysis of microalgae, marine diatoms and freshwater chlorophytes (Baumeister et al., 2019). Matrix-free nanophotonic ionization of analytes on Silicon Nanopost Array chip followed by MSI offers an effective platform for detection of small metabolites at single cell level (Walker et al., 2012; Korte et al., 2016). Desorption Electrospray Ionization is another soft ionization technique having capability to ionize wide variable molecules however poor spatial resolution is a limitation of this technique which can be improved further for SCM analyses. MS coupled with Microfluidic chips or microarray systems and 3D MALDI Mass Spectrometry Imaging are the other potential SCM techniques which can be used for analyzing virus-host interaction in near future. Single-Molecule Array which resembles the digital ELISA format may also be employed for targeted SCM by using specific tagged antibody against the target metabolite(s) (Minakshi et al., 2019a).

## Metabolomic Insight at Virus-Host Interface to Combat Emerging Viruses

Zika virus, Dengu virus, Chikungunya virus, HIV, avian influenza, Nipah (NiV) viruses, Ebola and Marburg filoviruses, severe acute respiratory syndrome (SARS) and Middle East respiratory syndrome coronavirus (MERS CoV) are the emerging viruses as evidenced in recent times (Afrough et al., 2019). Several of them are having zoonotic importance and emerge from viral spill over from the sylvatic cycle infecting multiple hosts. Metabolomic intervention can trace out some common metabolic pathway(s) in different host range which are also essential for viral replication (Passalacqua et al., 2018; Mayer et al., 2019). For Example, Progression of human cytomegalovirus (HCMV) infection has increased the metabolite flux through glycolysis, citric acid cycle, and pyrimidine nucleotide biosynthesis in human fibroblasts, probably to meet the energy demand for viral replication and supply of macromolecular precursors (Munger et al., 2008). HCMV mediated metabolic reprogramming also includes enhanced lipogenesis through mTOR-sterol regulatory element-binding protein (SREBP)-regulated and other pathways inducing several enzymes such as acetyl-CoA carboxylase, fatty acid synthase, fatty acid elongases etc. The synthesized very long chain and long chain fatty acids are essential for viral envelope production, because inhibition of such pathways result into impaired viral replication. Similarly other DNA viruses such as herpes simplex virus-1 (HSV-1), Kaposi’s sarcoma-associated herpes virus (KSHV), vaccinia virus (VACV) are also found to modulate glycolysis, glutamine metabolism and fatty acid synthesis as common pathways. Thus any substrate analog (e.g., glucose analog 2-deoxyglucose) or enzyme inhibitor which prevents such virus-induced host metabolic reprogramming can be effective against multiple viruses. Although RNA viruses like rhinovirus, hepatitis C virus and several emerging arboviruses such as Zika virus, Dengu virus, Chikungunya virus etc. modulate host metabolic pathways in a little different manner but glycolysis, glutamine metabolism and fatty acid synthesis remain as the prime targets. Similar host enzyme machineries are being exploited by these RNA viruses too. These common metabolic pathway nodes can be exploited for preventive, diagnostic and therapeutic management of emerging viral infections. Further, such exploration can be of interest for therapeutic repurposing of existing drugs to combat any emerging viral outbreak. For example, Remdesivir (GS-5734) is a novel nucleoside analog antiviral prodrug, actually developed for the treatment of single stranded RNA containing Ebola virus disease and Marburg virus infections, has now been suggested as therapeutic option against recent SARS-CoV-2 pandemic. SARS-CoV-2 is also having positive-sense single-stranded RNA genome and Remdesivir gets incorporated into the nascent viral RNA chains leading to inhibition of viral replication through premature termination of RNA transcription (Centers for Disease Control and Prevention, 2020; Liu et al., 2020). However, the toxic effects of such substrate analogs or pathway inhibitors on host cells should be critically analyzed prior to their extensive application. Recently, traditional anti-parasitic drug ivermectin has also found to exhibit *in vitro* antiviral activity against SARS-CoV-2 infection and warrants rapid further *in vivo* introspection to combat current COVID-19 pandemic. This is also an example of drug repurposing relying upon the virus-exploited common pathways. Because in earlier research, ivermectin has depicted efficacy against multiple RNA viruses such as DENV 1-4, West Nile Virus, influenza virus, Venezuelan equine encephalitis virus (VEEV) and human immunodeficiency virus-1 (HIV-1) by destabilizing the importin (IMP) α/β1 heterodimer and blocking IMP α/β1-mediated nuclear import of viral proteins (Caly et al., 2020).

Metabolite profiling can also provide an idea about the disease severity and predictive outcome of the viral infection. Serum metabolite analyses of H7N9 infected subjects have revealed that increase in palmitic acid, erucic acid, and phytal level is related to virus-induced repression of fatty acid metabolism and negatively correlated with the disease outcome (Sun et al., 2018).

In arboviral infections, metabolic reprogramming in arthropod vector and vertebrate host takes place in different manner. Zika virus (ZIKV) induced metabolic reprogramming of human foreskin fibroblast cells (HFF-1) shapes differently from C6/36 cells of *Aedes albopictus* mosquito which serves as the vector of ZIKV infection. Glucose influx has been increased through glycolysis in both the cases, however, it is mainly diverted toward tricarboxylic acid (TCA) cycle and amino acid synthesis in human cells whereas glucose contribution to TCA cycle gets reduced to augment the intermediates of pentose phosphate pathway in mosquito cells. Further, ZIKV infection depletes ATP level in HFF-1 cells leading to elevated AMPK phosphorylation and increased caspase-mediated cell death whereas neither such increase in AMP/ATP and ADP/ATP ratios as well as AMPK phosphorylation nor decreased cell viability has been evidenced in infected C6/36 mosquito cells. Such difference in cell viability may be contributed by both altered pentose phosphate pathway and AMPK activation (Thaker et al., 2019). So metabolic introspection is not only capable of elucidating virus-host interaction but also provides information regarding maintenance and propagation of virus through their arthropod vectors. Understanding of lifecycle of such viruses exploiting metabolic pathways within arthropod vectors can assist to design strategies for preventing their transmission in vertebrate hosts.

Considerable numbers of researches have followed such path providing metabolomic insight at virus-host interface. Chromatography (LC/GC) coupled with tandem mass spectrometry (MS/MS) is the most common method employed for metabolomics introspection followed by NMR, FTIR and fluorescence based methods (Table 1). Most of these experiments have reached upto the level of cell line but significant penetration at single cell level is yet to be achieved in terms of SCM analysis in the context of virus-host interaction. But single-cell analysis may elucidate far precise information regarding viral replication and infection kinetics which is beyond the capacity of population study (Guo et al., 2017). A high-throughput, microfluidics-based platform has been devised to perform kinetic analysis of green fluorescence protein (GFP) tagged polio virus (PV) infections in individual HeLa S3 cells. Monitoring of fluorescence intensity has revealed marked variation in viral replication and infection cycle in each of the GFP-PV infected cells which are uniquely and independently controlled by viral as well as the cellular factors. The time of onset of viral replication varied significantly among the individual infected cells. Similar cellular-factor regulated variable pattern has continued to follow throughout the viral infection cycle upto the cell lysis. The analysis at single cell level has unearthed plethora of valuable information regarding virulence determinants and mechanisms of drug action which have been escaped in population methods (Guo et al., 2017). The mentioned evidence is extremely useful to justify the value of single cell techniques over population measurements and several such SCM introspection at virus-host interface may follow the path in near future. It will not only surmount the heterogeneity issue of samples otherwise succumb to superimposed results but also help to identify the effect of mutation both in virus as well as host, virus homing affinity toward cell types, post-infection survival, progression of disease etc. Although SCM analyses have its own advantage but it must be complemented by the bulk metabolomic analyses of the infected tissue or organ because cell-to-cell communication may have crucial effect in virus infection cycle which may not be reflected in SCM analyses. Thus a combinatorial approach is more beneficial over a standalone SCM introspection so that the real reflection of cellular heterogeneity at virus-host interface as well as the influence of cell communication can be elucidated precisely. The specific information extracted from such analyses like mutation study along with computational modeling may assist in future vaccine formulation, mapping of virus evolution strategy to identify the potential hot-spots of novel viral emergence, their virulence and putative contentment recipes.

However, the inherent challenges of SCM techniques require to be surmounted with diligence and passion for proper utilization of the potential of single-cell analyses. The difficulty in single cell isolation along with rapid turnover, transport and degradation of the cellular metabolites are the key challenges of SCM analyses. Rapid metabolite turnover can produce random noise which may create analytical error and difficulty in differentiating infected cells from the healthy one. Thus normalization of such random error must be performed during the experimental set-up for SCM data acquisition to differentiate the deterministic effects from the stochastic one. Similar type of random fluorescence noise normalization has been performed by Guo et al. (2017) during GFP-PV infected single-cell analyses. Further dealing with minute sample volume, diverse types of metabolites and their femto-molar range concentration demands high sensitivity and throughput of the analytical platform which is essential particularly for SCM analyses. For example, NMR is extremely robust, reliable, requiring minimal sample processing and commonly employed metabolomics analysis platform for population study but low sensitivity has limited the utility of the technique for SCM analyses. Proper analyses of SCM data is another crucial challenge which has been eased out significantly with recent advances in bioinformatics tools and growing metabolite database (Minakshi et al., 2019a). Moreover, integration of SCM with single-cell proteomics, transcriptomics, genomics and interactomics can provide a whole-some image of the life processes which may assist to explore the biological missing links regarding virus infection and transmission cycle.

## Conclusion

Rapid development in single cell isolation and handling methods, persistent improvement in sensitivity and resolution of the analytical platforms along with growing database and data interpretation programs are smoothening the hurdles of SCM to introduce it at the virus-host interface as early as possible. The SCM derived precise information regarding the altered metabolism of infected cell over the healthy cells may assist to investigate the virulence, survival, and progression of diseases. The exploration of cellular heterogeneity can illuminate the aspects which remain concealed in population study. Thus SCM holds the promise as a key tool of future to introspect virus-host interaction and assist in efficient management of emerging viral disease.

## Author Contributions

MP and MG conceptualized the manuscript. RK and SK wrote the manuscript. MP critically analyzed and improved the manuscript.

## Conflict of Interest

The authors declare that the research was conducted in the absence of any commercial or financial relationships that could be construed as a potential conflict of interest.
